# In Situ Study of the Interface-Mediated Solid-State Reactions during Growth and Postgrowth Annealing of Pd/a-Ge Bilayers

**DOI:** 10.1021/acsami.2c20600

**Published:** 2023-02-15

**Authors:** Bärbel Krause, Gregory Abadias, David Babonneau, Anny Michel, Andrea Resta, Alessandro Coati, Yves Garreau, Alina Vlad, Anton Plech, Peter Wochner, Tilo Baumbach

**Affiliations:** † Institut für Photonenforschung und Synchrotronstrahlung (IPS), Karlsruher Institut für Technologie, D-76021 Karlsruhe, Germany; ‡ Institut Pprime, Département Physique et Mécanique des Matériaux, UPR 3346 CNRS, Université de Poitiers, SP2MI, TSA 41123, Cedex 9 86073 Poitiers, France; § Synchrotron SOLEIL, L’Orme des Merisiers, Départementale 128, 91190 Saint Aubin, France; ∥ Laboratoire Matériaux et Phénomenes Quantiques, Université Paris Cité, 75013 Paris, France; ⊥ Max Planck Institute for Solid State Physics, Heisenbergstraße 1, D-70569 Stuttgart, Germany; # Laboratorium für Applikationen der Synchrotronstrahlung (LAS), Karlsruher Institut für Technologie, D-76021 Karlsruhe, Germany

**Keywords:** in situ monitoring, X-ray, curvature measurement, germanides, sputter deposition, solid-state reaction, local epitaxy, Schottky contact

## Abstract

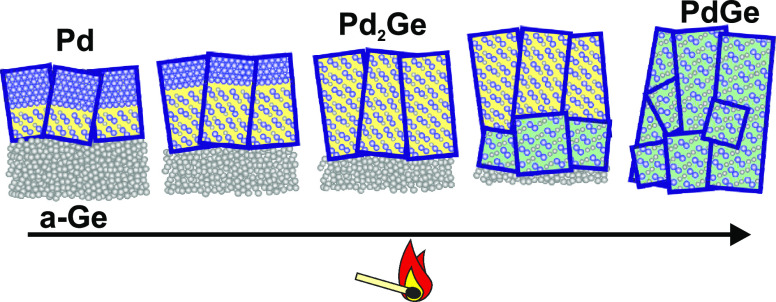

Ohmic or Schottky contacts in micro- and nanoelectronic devices are formed by metal–semiconductor bilayer systems, based on elemental metals or thermally more stable metallic compounds (germanides, silicides). The control of their electronic properties remains challenging as their structure formation is not yet fully understood. We have studied the phase and microstructure evolution during sputter deposition and postgrowth annealing of Pd/a-Ge bilayer systems with different Pd/Ge ratios (Pd:Ge, 2Pd:Ge, and 4Pd:Ge). The room-temperature deposition of up to 30 nm Pd was monitored by simultaneous, in situ synchrotron X-ray diffraction, X-ray reflectivity, and optical stress measurements. With this portfolio of complementary real-time methods, we could identify the microstructural origins of the resistivity evolution during contact formation: Real-time X-ray diffraction measurements indicate a coherent, epitaxial growth of Pd(111) on the individual crystallites of the initially forming, polycrystalline Pd_2_Ge[111] layer. The crystallization of the Pd_2_Ge interfacial layer causes a characteristic change in the real-time wafer curvature (tensile peak), and a significant drop of the resistivity after 1.5 nm Pd deposition. In addition, we could confirm the isostructural interface formation of Pd/a-Ge and Pd/a-Si. Subtle differences between both interfaces originate from the lattice mismatch at the interface between compound and metal. The solid-state reaction during subsequent annealing was studied by real-time X-ray diffraction and complementary UHV surface analysis. We could establish the link between phase and microstructure formation during deposition and annealing-induced solid-state reaction: The thermally induced reaction between Pd and a-Ge proceeds via diffusion-controlled growth of the Pd_2_Ge seed crystallites. The second-phase (PdGe) formation is nucleation-controlled and takes place only when a sufficient Ge reservoir exists. The real-time access to structure and electronic properties on the nanoscale opens new paths for the knowledge-based formation of ultrathin metal/semiconductor contacts.

## 1 Introduction

Interdiffusion and solid-state reactions at the metal–semiconductor interface are of particular importance for the design of microelectronic devices. Sometimes, they are undesired, reducing, e.g., the performance of optoelectronic devices.^1^ Many applications, however, rely on interface-mediated solid-state reactions as key element of the manufacturing process.^2,3^ Among other applications, metal–semiconductor interfaces are employed as ohmic (nonrectifying) or Schottky (rectifying) contacts in Si- and Ge-based devices. Compared to silicon, germanium has a smaller band gap and a higher charge carrier mobility, promising faster electronic devices. The larger exciton radius in Ge compared to Si facilitates the generation of quantum states in Ge. This explains the recent interest in Ge-based devices such as integrated avalanche photodetectors,^4,5^ nanowire electronics,^6,7^ quantum computing,^8^ plasmonic detectors and waveguides,^9−11^ hydrogen sensing,^12^ and printable electronics.^13,14^

The electronic properties of a metal–semiconductor interface depend on the Schottky barrier that is only weakly influenced by different elemental metals. This phenomenon is called Fermi-level pinning. For silicides and germanides, a much larger variation was observed,^15^ opening different pathways for device layout. Metal–semiconductor compounds are often formed by solid-state reactions. The advantage of solid-state reactions is their simple realization by postgrowth thermal treatment of metal–semiconductor bilayer systems. Nevertheless, the control of the phase and microstructure formation during annealing remains challenging. The lack of control on the atomic scale is unsatisfactory since the Schottky barrier depends on the specific atomic arrangements close to the interface.^16−18^

**Figure 1:**
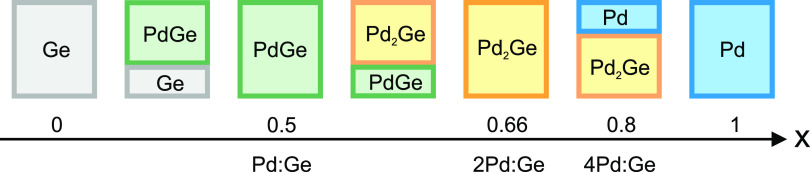
Phases expected after annealing of Pd/a-Ge bilayers with different Pd fractions *x*, referring to the average atomic composition Pd_*x*_Ge_1–*x*_ of the bilayer. The experimentally studied Pd:Ge ratios are indicated.

Over the past several decades, numerous experimental studies have advanced the knowledge about interface-mediated phase formation processes during annealing of metal/semiconductor systems, identifying several aspects which are crucial for phase formation: the thermally activated diffusion through the interface, the competition between diffusion and nucleation processes, and the atomic reservoir.^19−22^ The atomic reservoir determines the equilibrium composition assumed at sufficiently high temperatures and reaction times, as schematically shown in Figure 1 for the Pd–Ge system. The phase and microstructure evolution during annealing are affected by the as-deposited interface, which can be manipulated, e.g., by deposition of diffusion barriers,^1,23,24^ or controlled defect formation via ion bombardment.^25,26^ Modification of the interface during growth, choosing appropriate deposition parameters, is the least invasive approach, but needs a detailed understanding of the interplay between as-deposited metal/semiconductor interface and structure formation during subsequent solid-state reaction.

Obtaining nanoscale information about metastable structures is extremely challenging. The low energy required for the interface reaction complicates the interpretation of high-resolution transmission electron microscopy results commonly used to study buried interfaces, facilitating intermixing, compound formation, amorphization, and selective sputtering during sample preparation and electron irradiation.^27−30^ Noninvasive real-time methods can overcome this problem. Furthermore, they provide real-time access to the evolving structures. Reflection high-energy electron diffraction is commonly used for monitoring the structure formation during molecular beam epitaxy. However, the method is only compatible with sputter deposition processes when using differential pumping,^31^ which affects the growth conditions significantly. X-ray methods do not suffer from this limitation, and are therefore well established for monitoring industrially relevant sputter deposition processes.^32−35^ We have combined real-time synchrotron experiments during magnetron sputter deposition and annealing of Pd/amorphous germanium (a-Ge) bilayers. The real-time information during deposition of Pd/a-Ge was obtained using simultaneous X-ray methods and stress monitoring.^36,37^ The structure formation during subsequent annealing was studied by real-time X-ray diffraction (XRD). Complementing the real-time studies, electronic state, chemical composition, and morphology of the as-deposited and annealed samples were characterized by X-ray photoelectron spectroscopy (XPS) and noncontact atomic force microscopy (AFM). All experiments described above were performed in situ; i.e., throughout the entire study the samples were kept under ultra-high-vacuum (UHV) conditions to avoid contamination-induced reactions.

The present study builds upon recent results for Pd/a-Si: using the above-described methodology, we have established a detailed picture of the Pd/a-Si interface formation during deposition.^37^ The initially forming amorphous Pd_2_Si crystallizes at a critical thickness, resulting in a surprisingly well-oriented Pd_2_Si interlayer which serves as the template of the subsequently deposited, highly oriented Pd(111) film. Compared to crystalline substrates, a laterally isotropic, amorphous semiconductor layer reduces the number of competing processes, thus simplifying the fundamental understanding of the structure formation without affecting the phase sequence. Interestingly, Pd/Si^38−40^ and Pd/Ge bilayers^41−43^ share a similar phase formation sequence during annealing. The relevant crystal structures are summarized in Table S1 (Supporting Information). Most authors agree that the first forming phase is hexagonal Pd_2_Ge (Pd_2_Si), followed by orthorhombic PdGe (PdSi). For practical reasons we have selected Pd–Ge, the material system with lower phase transition temperatures, as the model system for the interface-mediated phase formation during growth and subsequent annealing. The Pd-induced crystallization of a-Ge is outside the studied temperature range.^44,45^

In the following, we address three main issues: (1) Does the analogy between the material systems Pd–Ge and Pd–Si extend to the interlayer formation mechanism? (2) How is the structure formation during annealing of Pd/a-Ge related to the structure of the as-deposited layer system? (3) Which impact does the available material reservoir have on the structure formation during annealing? Beyond the fundamental understanding of structure formation, our study gives access to the interplay between structure and electronic properties during contact formation, and provides insight into structural aspects which affect the long-term contact stability.

## 2 Experimental Section

### 2.1 Sample Preparation

All deposition and annealing procedures were performed in a modular sputter chamber designed for real-time X-ray studies under UHV conditions (base pressure 2 × 10^–6^ Pa).^46^ Pd/a-Ge bilayer systems with layer thicknesses in the nm range were deposited at room temperature by magnetron sputtering on silicon wafers covered by native oxide. For the real-time experiments, thin substrates with a size of 9 × 11 × 0.1 mm^3^ were used. Due to the requirements of the curvature measurements, these samples were not clamped to the substrate holder. UHV surface characterization was performed on 20 × 20 × 1 mm^3^ substrates clamped to the substrate holder. The target–substrate distance was 325 mm for Pd and 129 mm for Ge. Pd was deposited at a deposition rate *F*_Pd_ = 0.020 ± 0.001 nm/s, using a DC power of 40 W. The deposition rate of Ge at an RF power of 30 W was *F*_Ge_ = 0.0495 ± 0.002 nm/s. For sample I, the Ge buffer layer was deposited at 60 W (*F*_Ge_ = 0.140 ± 0.002 nm/s). The change in deposition rate did not visibly influence the structure formation during deposition. The deposition rates were obtained from the real-time X-ray reflectivity (XRR) analysis (see Section 3.1). In the following, *h* indicates all thickness values derived from the deposition rates and times.

The in situ study during postgrowth annealing of as-deposited bilayers was done at a rate of 1.8 K/min up to the maximum temperature *T* = 600 K. The temperature evolution at the sample surface was verified by reference measurements with a thermocouple mounted on the sample holder. Note that the clamped reference samples had a slightly better thermal contact to the substrate holder.

Table 1 gives an overview over the samples discussed in Section 3. The real-time growth study is based on the samples I and Ib. Assuming the crystal structures summarized in Table S1, the formation of 1 nm Pd_2_Ge (PdGe) requires 0.66 nm (0.47 nm) Pd and 0.51 nm (0.72 nm) Ge. The in situ study during postgrowth annealing is based on the unclamped samples II–IV with different Pd:Ge ratio but the same expected Pd_2_Ge thickness of approximately 13.5 nm. Complementary UHV surface characterization after deposition was performed on the clamped samples II*, III*, and V*-XVIII*. The samples II* and III* were also characterized after annealing.

**Table 1:** Deposition Time *t*_Ge_ (*t*_Pd_) and Corresponding Layer Thickness *h*_Ge_ (*h*_Pd_) for the Magnetron Sputter-Deposited Pd/Ge Bilayers^a^

sample	*t*_Ge_	*t*_Pd_	*h*_Ge_	*h*_Pd_	crystalline phases after annealing
(Pd:Ge ratio)	(s)	(s)	(nm)	(nm)	
I	138	500	19.3	10.0	
Ib	138	1500	6.8	30.0	
					
II (Pd:Ge)	276	450	13.6	9.0	PdGe and Pd_2_Ge
III (2Pd:Ge)	138	450	6.8	9.0	Pd_2_Ge
IV (4Pd:Ge)	138	900	6.8	18.0	Pd and Pd_2_Ge
					
II* (Pd:Ge)	276	450	13.6	9.0	PdGe
III* (2Pd:Ge)	138	450	6.8	9.0	Pd_2_Ge
V*-XVIII*	138	60–300	6.8	1.2–6.0	

a The samples are sorted according to the experiment: I and Ib, combined real-time experiments during deposition; II–IV, real-time experiments during deposition and annealing; II*, III*, and V*-XVIII*, UHV surface characterization. For the annealed samples, the nominal Pd:Ge ratio and the phases observed after annealing are indicated.

### 2.2 Real-Time Characterization

The results presented in this paper were obtained during two synchrotron experiments. The experiment at the synchrotron SOLEIL was optimized for monitoring highly oriented thin films during deposition. The second experiment, performed at the KIT light source, combines real-time observations during growth and subsequent annealing of the same sample but was less sensitive to narrow textures. All X-ray measurements were performed at incidence angles well above the critical angle for total external reflection; i.e., the XRD signal provides information about the entire film thickness.

**Figure 2:**
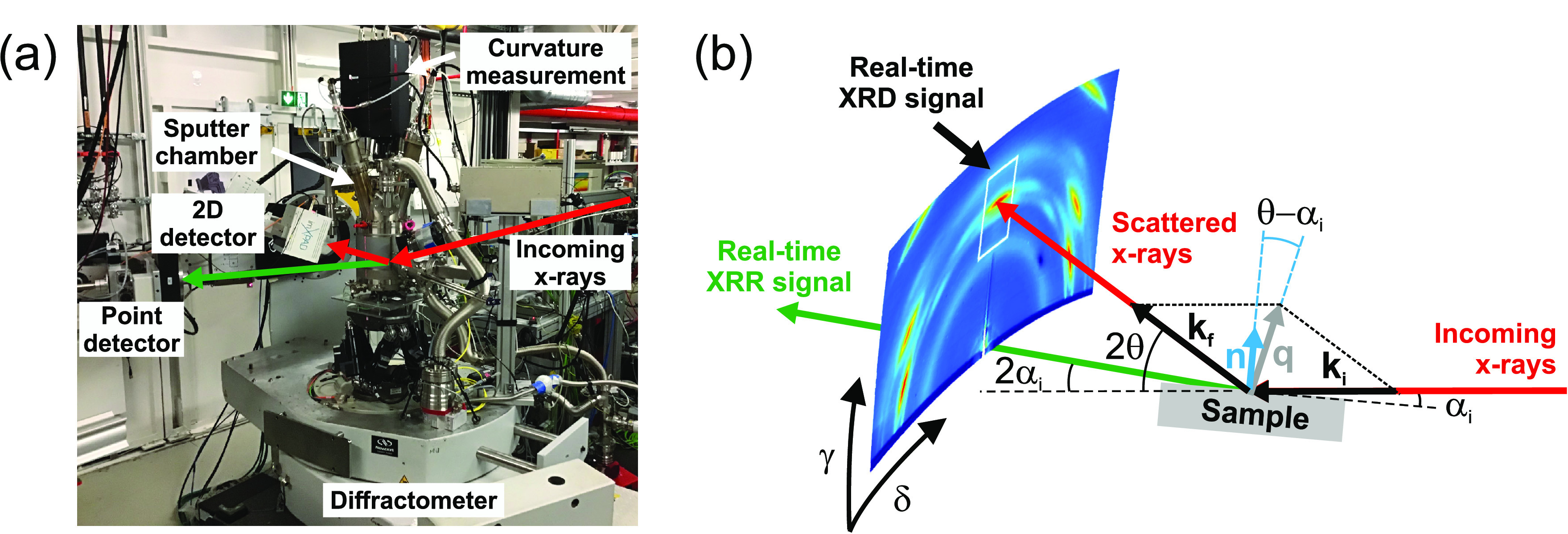
(a) Experimental setup for the real-time study at the SIXS beamline (SOLEIL) and (b) grazing incidence scattering geometry with the incident angle α_i_. The reflected X-ray beam is indicated. The diffraction geometry is shown for the main Pd(111) Bragg reflection at the Bragg angle 2θ. Due to the grazing incidence geometry, the momentum transfer *q* = *k*_f_ – *k*_i_ is oriented at the angle θ – α_i_ to the surface normal *n*. *k*_i_ and *k*_f_ are the wave vectors of the incoming and the scattered X-ray beam. The XRD map is obtained by scanning the detector angles δ and γ. During deposition, the 2D detector monitors the region indicated by the white rectangle.

Following the experimental approach described in refs (36) and (37), simultaneous XRR, XRD, and optical curvature measurements during deposition were performed at the SIXS beamline of the synchrotron SOLEIL (France). The experimental setup is shown in Figure 2a; the scattering geometry is schematically shown in Figure 2b. The incoming X-ray beam with a photon energy *E* = 15 keV, a beam size of 0.05 × 1.5 mm (*v* × *h*), and an incident angle α_i_ = 1.6° had a footprint of 1.8 mm. The XRR signal at fixed angle was recorded with a point detector at 1100 mm distance and a slit size of 1 × 2 mm^2^. The real-time XRR signal is sensitive to the time-dependent scattering length density (SLD) profile which is directly related to the electron density. The SLD was parametrized using a layer model, where each layer is described by its bulk electron density, a time-dependent thickness *D*^XRR^, and a time-dependent interface roughness σ. The XRR intensity was calculated from the SLD profile using the Parratt algorithm. The roughness parameters and the deposition rates of the growing layers were fitted. A detailed description of the fit approach and its application to Pd/a-Si can be found in ref (37).

The XRD signal was collected with a 2D detector (XPAD) at a distance of 332 mm. Both detectors were mounted with an angular separation of 22° on the same detector arm. Large-area XRD maps were obtained by scanning the angular detector positions. The measured intensity distribution is expressed in angular coordinates δ and γ (horizontal and vertical angle) with respect to the direct beam, or in reciprocal space coordinates  with *q*_∥_ in the sample plane and *q*_*z*_ along the surface normal. Due to the grazing incidence geometry, the region close to (0, *q*_*z*_) is not accessible.

The bilayers were deposited on thin Si wafers with 100 ± 2 μm thickness. The curvature of the wafers changes during deposition, giving information about the stress evolution.^47^ This complementary information was measured with a multiple-beam optical stress sensor (*k*-space associates). The change of the sample curvature, Δκ, was determined from a pattern of 3 × 3 laser spots. From this, the film force per unit width (*F*/*w*) was calculated using the Stoney equation. *F*/*w* is equivalent to the stress × thickness product, σ × *h*. By convention, positive (negative) derivatives of *F*/*w* vs *h* curves correspond to tensile (compressive) incremental stress.

The second real-time study was performed at the MPI beamline of the KIT synchrotron, using a similar setup. The incoming X-ray beam with the beam size 0.07 × 2.0 mm^2^ and *E* = 10 keV had a footprint of 2.0 mm at α_i_ = 2°. The diffracted intensity was recorded with a 2D detector (Eiger) at a distance of 230 mm. The angle between both detectors was 24°. During annealing, line scans along δ were performed with the 2D detector to cover a larger region of reciprocal space. Each line scan took 1 min. After 10 line scans, the sample height was adjusted to compensate for the thermal expansion of the heating station. For a deeper understanding of the phase transformation mechanism, radial scans at different angles χ to the surface normal were extracted from the real-time XRD maps. The grain size *D*^XRD^ of the different phases was determined using the full width at half-maximum (fwhm) Δ*q* of the Bragg peaks and the relation *D*^XRD^ = 2π/Δ*q*. The fwhm of the Bragg peaks was determined by simple peak fits of the radial scans. After initial tests with various models, the Pd peak was fitted with a Lorentzian, and the germanide peaks were fitted with a pseudo-Voigt function (Gaussian fraction fixed to 0.7). In addition to the real-time XRD measurements, specular XRD scans after annealing were performed ex situ, using a Rigaku Smartlab system with a Cu rotational anode in parallel-beam geometry. The Cu K_β_ line was suppressed by a Ni filter.

### 2.3 UHV Surface Characterization

For the XPS and AFM measurements, the growth chamber was docked to a UHV cluster system, allowing for in-vacuum transfer of the samples to the respective analysis chambers. The XPS measurements were performed with a non-monochromatized Mg K_α_ source and a Phoibos 150 analyzer (Specs), calibrated with a silver reference foil. Survey and high-resolution spectra were recorded with a pass energy of 50 and 20 eV, respectively. Noncontact AFM measurements were performed with a large-sample scanning probe microscope (Scienta Omicron). To verify the interpretation of the results, AFM images were recorded at several sample positions and with different scan sizes in the range from 250 × 250 nm^2^ to 2 × 2 μm^2^.

## 3 Results

In the following, we present the results obtained during deposition and postgrowth thermal treatment of Pd on a-Ge, connecting the fundamental processes governing the phase and structure evolution during interface formation and the subsequent solid-state reaction.

### 3.1 Isostructural Interface Formation of Pd/a-Ge and Pd/a-Si

**Figure 3:**
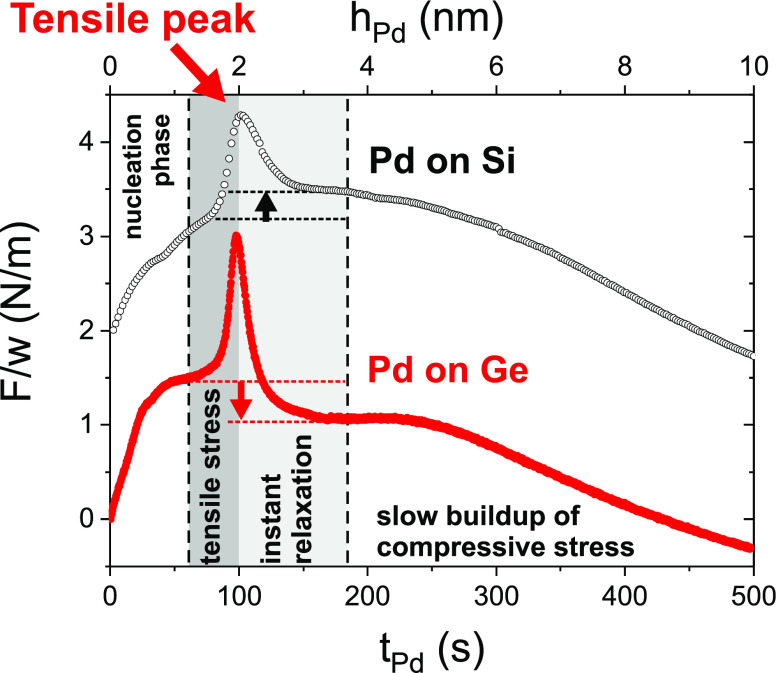
Real-time stress evolution during deposition of Pd on a-Ge (sample I, red dots) and a-Si (black circles, plotted with a vertical offset). Dashed lines mark the tensile peak which is defined by a sudden stress increase (dark gray background) and subsequent stress release (light gray background). The relative change of stress state before and after tensile peak (indicated by arrows) is characteristic for the semiconductor element. Partially reproduced with permission from ref (37). Copyright 2019 American Chemical Society.

To introduce the structure formation near the metal/semiconductor interface, Figure 3 compares real-time *F*/*w* data for up to 10 nm Pd deposition on a-Ge (red dots) and a-Si (black circles). The general evolution of both curves is similar: a tensile peak occurs after deposition of *h*_Pd_ = 2.00 nm (*t*_Pd_ = 100 s), followed by instant relaxation and a slowly increasing compressive stress during later growth. We have verified experimentally that the position of the tensile peak is reproducible within ±2 s. For Pd/a-Si, the tensile peak has been associated with the crystallization of an amorphous Pd_2_Si interlayer formed during the very first stages of Pd deposition. The compressive stress during later growth was attributed to Pd crystal growth.^37^

**Figure 4:**
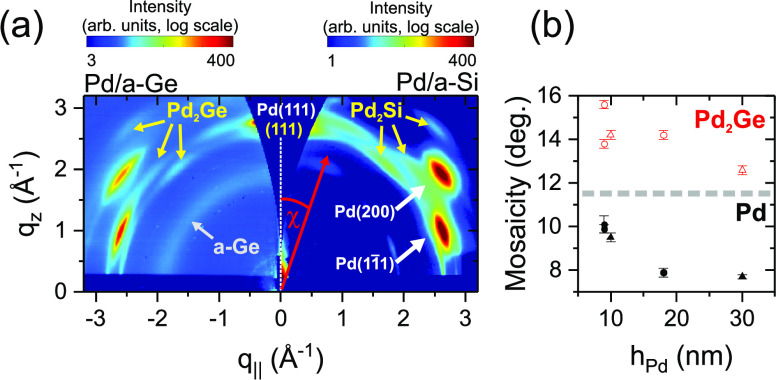
(a) Postgrowth reciprocal space maps of sample I (left panel) and 16.5 nm Pd on 4.6 nm a-Si (right panel), revealing the structural similarities between the crystalline interlayers. For quantitative analysis, radial scans were extracted at different angles χ with respect to the surface normal. (b) Mosaicity of Pd_2_Ge (open symbols, red) and Pd (black symbols), extracted from Pd/a-Ge reciprocal space maps with different amounts of Pd. The samples were studied at the synchrotron SOLEIL (triangles, samples I and Ib) and the KIT synchrotron (circles, samples II–IV). Reprinted in part with permission from ref (37). Copyright 2019 American Chemical Society.

In analogy to Pd/a-Si, the real-time stress data suggest the formation of an isotypic Pd_2_Ge interlayer. This is confirmed by the postgrowth XRD signal shown in Figure 4a: The left panel shows the XRD pattern of sample I and the right panel the XRD pattern of 16.5 nm Pd on 4.6 nm a-Si. The intensity distribution of both samples is similar, with intense Pd peaks (labeled in white), and weaker Pd_2_Ge or Pd_2_Si peaks (e.g., the peaks indicated by yellow arrows). These results therefore suggest that Pd(111) grows on top of a Pd_2_Ge(111) interlayer with a thickness of at least  nm (assuming bulk densities and the reaction of *h*_Pd_ = 2.00 nm). In agreement with this, a Pd_2_Ge grain size of  nm in vertical and at least 10 nm in lateral directions was calculated from the fwhm of the diffraction rings. The orientation distribution of the crystallites was determined from the peak width along the diffraction ring (Figure 4b). The mosaicity of the interlayer (open symbols, red) is in the range 14 ± 2° and does not show any systematic variation with the deposited Pd amount. The Pd mosaicity (black symbols) is significantly lower and decreases with increasing Pd thickness, indicating competitive crystal growth.

**Figure 5:**
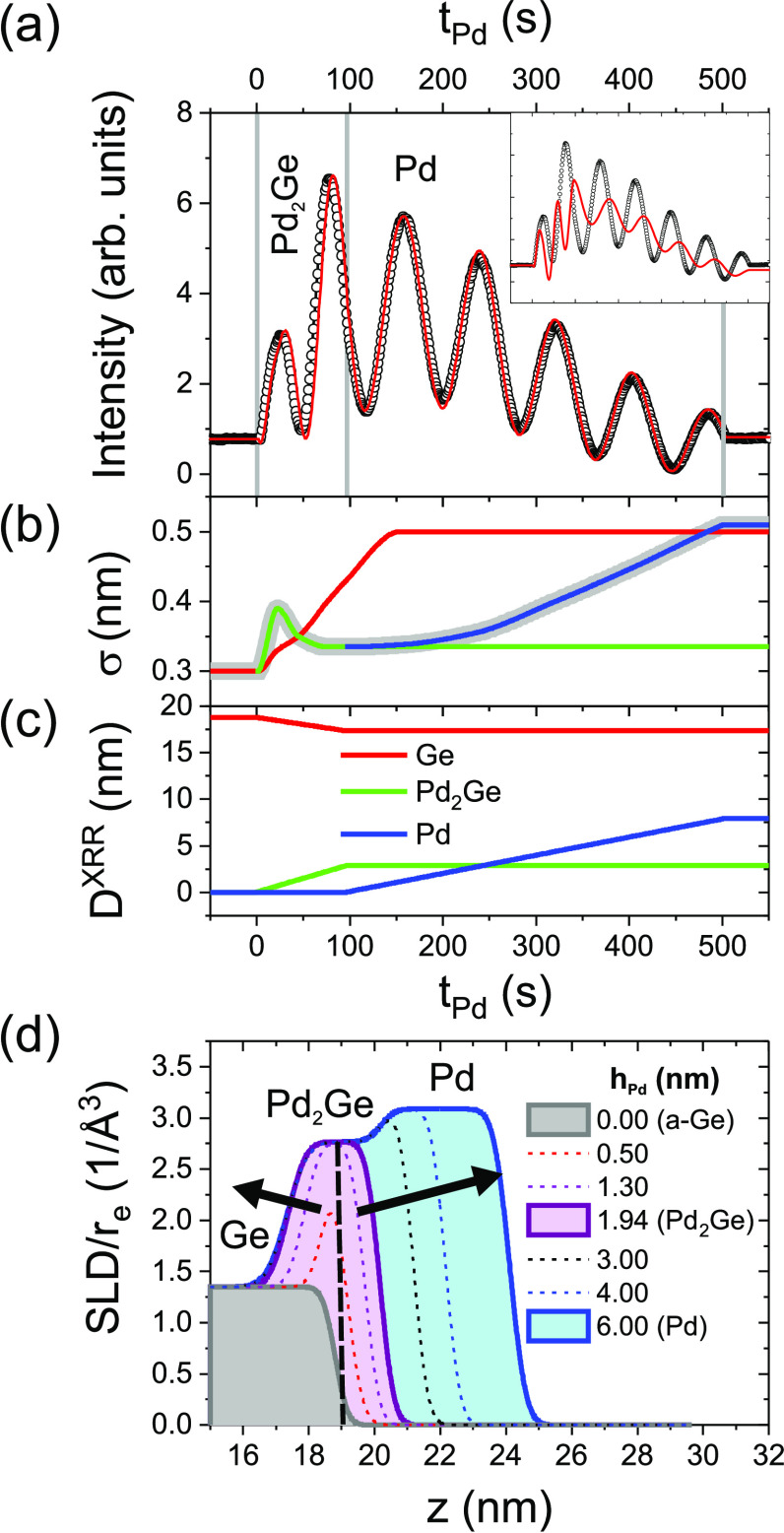
(a) Experimental (black dots, sample I) and simulated (red line) XRR intensity during Pd deposition on a-Ge. (b, c) Fit parameters (roughness and layer thickness) of Ge (red), Pd_2_Ge (green), and Pd (blue). The roughness of the topmost layer is marked by a wide line (gray). (d) Time-dependent scattering length density (SLD) profile. *r*_e_ is the electron radius; *z* is the distance from the substrate surface. The vertical dashed line marks the position of the a-Ge surface at *t*_Pd_ = 0 s. The arrows indicate the evolution of the surface and a-Ge/Pd_2_Ge interface during Pd deposition. To demonstrate the experimental sensitivity, the inset in panel a shows a simulation assuming the formation of a PdGe instead of a Pd_2_Ge interlayer.

The real-time XRR signal (dots), shown in Figure 5a for sample I, gives information about the deposited material, independent of its crystallinity. The data were reproduced by modeling a time-dependent SLD profile (red line). The main assumptions of the fit model are a constant deposition rate of Pd, bulk densities for all layers, and the instant reaction of Pd and Ge to Pd_2_Ge during early growth stages, reducing thus the thickness of the a-Ge buffer layer. Figure 5b,c shows the manually optimized roughness and thickness profiles of the respective layers.

Figure 5d compares the resulting SLD profiles during early growth stages, normalized to the electron radius *r*_e_. The vertical dashed line marks the position of the a-Ge surface at *t*_Pd_ = 0 s. The arrows indicate the evolution of the Ge/Pd_2_Ge interface and the growth front. As seen in Figure 5a, the experimentally observed phase shift and change in oscillation frequency after the second oscillation maximum are reproduced assuming a transition from Pd_2_Ge to Pd at *t*_Pd_ = 97 s and an interlayer thickness  nm (close to ). Within the experimental uncertainty, the parameter ranges *t*_Pd_ = 97 ± 2 s and *F*_Pd_ = 0.020 ± 0.001 nm/s are compatible with the observed oscillation periods. The small phase shift between experimental and simulated oscillations indicates a systematic error of the order of 0.1 nm for . To demonstrate the sensitivity of the simulation on the layer model, the inset in Figure 5a shows a simulation with the same parameters but assuming PdGe instead of Pd_2_Ge formation. This model results in a different phase shift at *t*_Pd_ = 97 s, and much faster oscillations during interlayer formation.

The time-dependent roughness parameters describe the interface width during deposition, reflecting roughness changes and intermixing (Figure 5b). The experimental uncertainty of the roughness parameters is dominated by systematic errors. These are typically of the order of 0.01–0.02 nm. The surface roughness, σ_surf_ [wide line (gray)], is the roughness parameter of the topmost layer which is also accessible by AFM measurements. During interlayer formation, σ_surf_ remains close to the initial a-Ge roughness. As already reported for Pd/a-Si, σ_surf_ increases continuously during subsequent Pd growth (visible as damping of the XRR oscillations). The interlayer formation is accompanied by a significant increase of σ_Ge_ from 0.3 to 0.5 nm (extending even after reactive Pd_2_Ge growth), and a small peak of . Note that the linear increase of σ_Ge_ is likely a simplification. In the peak region, the assumption  of the layer model is not fulfilled, and σ_surf_ overestimates the roughness values expected from AFM measurements. The parameters describe an effective change of the SLD profile (dominated by interface broadening) which is attributed to interdiffusion at the buried Pd_2_Ge/a-Ge interface. We do not find any indication for interdiffusion at the Pd/Pd_2_Ge interface.

The XRD and XRR results confirm the analogy between Pd/a-Ge and Pd/a-Si. Surprisingly, the germanide formation stopped already at *t*_Pd_ = 97 s ( nm) while the silicide formation continued up to *t*_Pd_ ≈ 130 s, resulting in a much thicker interlayer with  nm.^37^ A closer look at the *F*/*w* evolution in Figure 3 reveals further differences between both material systems. For Pd/a-Ge, e.g., the tensile peak is narrower, indicating a faster strain relaxation after crystallization, and the offset between strain state before and after tensile peak (marked by an arrow) is tensile for Pd/a-Si but compressive for Pd/a-Ge.

### 3.2 Crystal Growth during Deposition

**Figure 6:**
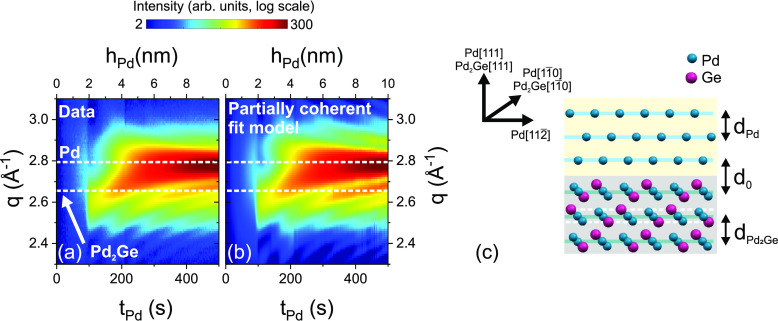
(a) Experimental and (b) simulated XRD intensity evolution during deposition (sample I, for details see the text and Supporting Information). Horizontal dotted lines indicate the expected Pd(111) and Pd_2_Ge(111) peak positions. (c) Pd/Pd_2_Ge bilayer model representing the structure of an individual crystalline grain. The grain is defined by the bulk lattice spacings of each phase, and the lattice spacing *d*_0_ in between the phases.

The real-time XRD data recorded during deposition of sample I (Figure 6a) provide further details about the crystallization process of Pd_2_Ge and Pd. The data were collected at the border of the inaccessible region shown in Figure 3 (dark region around *q*_∥_ = 0 Å^–1^). *q⃗* follows a slightly curved line which is a close fit to a radial scan at χ = 9°. At this angle, the signal is dominated by grains tilted by 9°. Due to the large mosaicity, the quantitative analysis is not affected by the slight curvature of the scan.

During initial growth, a broad and weak intensity distribution is observed. At *t*_Pd_ = 90 ± 2 s, a narrow peak close to the expected Pd_2_Ge(111) position occurs. This peak is attributed to the sudden (re)crystallization of the already formed amorphous or nanocrystalline Pd_2_Ge. During further deposition, the peak shifts continuously to the Pd(111) position. For *t*_Pd_ ≳ 200 s, an asymmetric Bragg peak with pronounced Laue oscillations is observed (see also Figure S1a of the Supporting Information). Similar intensity distributions are known, e.g., from strained epitaxial layers on single-crystalline substrates, but their observation for polycrystalline bilayers is surprising.

To reproduce the data (Figure 6b), a modified crystal truncation rod approach was developed. The model takes into account the crystal orientation along the surface normal, known from postgrowth XRD, and the time-dependent layer thickness obtained from the XRR data. Each individual crystallite of the polycrystalline film is assumed to consist of two layers, Pd(111) and Pd_2_Ge(111), separated by *d*_0_ (Figure 6c). Based on this model, two scattering contributions were assumed: the coherent, kinematic scattering from perfectly aligned Pd(111) and Pd_2_Ge(111) crystallites (needed only for *t*_Pd_ ≥ 90 s), and an incoherent contribution. The incoherent contribution represents the amorphous layer before crystallization, and a nonepitaxial fraction after crystallization. The amplitudes of the coherent and incoherent scattering contribution were allowed to vary freely. All other parameters were varied in a narrow range around the expected values (for more details, see the Supporting Information).

The calculated intensity distribution in Figure 6b reproduces all details of the experimental *q*–*t* map. Without assuming complex strain distributions, the peak shift with deposition time results from the interference between X-rays scattered by Pd_2_Ge and Pd (see also Figure S1a). After interlayer formation, the thickness of the Pd layer increases linearly with deposition time. The best fit was obtained with the distance *d*_0_ = (1 ± 0.1) × . As shown in Figure 6c, each Pd_2_Ge(111) lattice plane can be described by two sublayers containing Pd and Ge (dashed white lines) and a central sublayer containing only Pd. In this plane, the Pd atoms form triangles, matching the Pd arrangement in the (111) plane.^37^ With *d*_0_ = , the first Pd layer takes the position of the central Pd plane of the germanide, indicating a (local) epitaxial relation between Pd and Pd_2_Ge. However, it was not possible to distinguish between different Pd_2_Ge terminations at the interfaces.

A locally epitaxial relationship between silicide and Pd was already proposed for Pd/a-Si, but not yet experimentally confirmed. The coherent scattering of germanide and Pd provides evidence for a well-defined structural relationship at the Pd(111)/Pd_2_Ge(111) interface. The germanide unit cell is slightly larger than the silicide unit cell (Table S1), resulting in a lattice mismatch of 5.6% between Pd_2_Ge  and Pd , while the mismatch between Pd_2_Si  and Pd  is only 2.2%. Therefore, the growing Pd layer exerts a higher compressive force on the underlying Pd_2_Ge interlayer. The epitaxial lattice strain contributes to the offset of *F*/*w* before and after the tensile peak (marked by arrows in Figure 3), which is compressive for Pd/a-Ge but tensile for Pd/a-Si.

### 3.3 Thickness-Dependent XPS Study of the Interlayer Formation during Deposition

**Figure 7:**
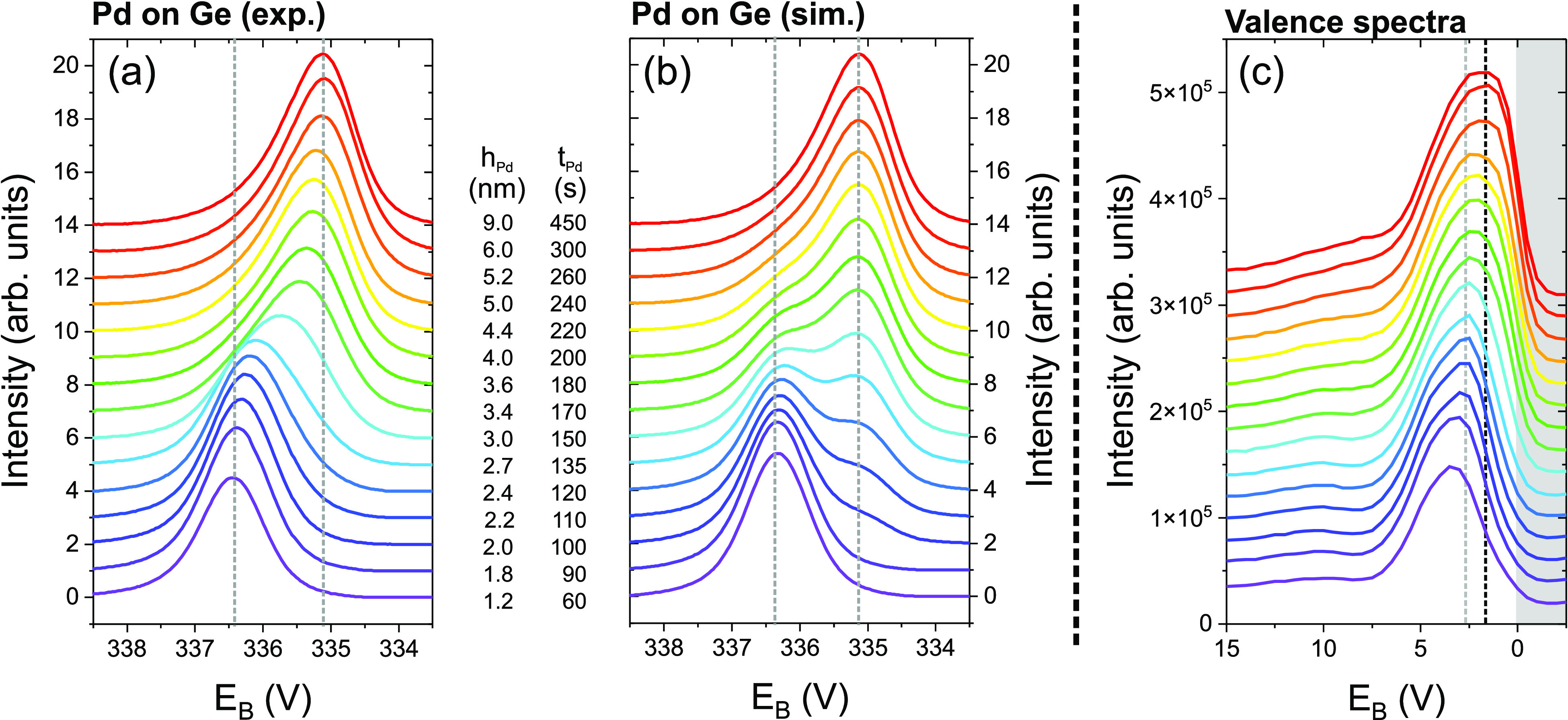
(a) Experimental (samples III*, V*-XVIII*) and (b) simulated evolution of the Pd 3d_5/2_ XPS signal during deposition of Pd on a-Ge. The XPS spectra are vertically shifted for different *h*_Pd_ (*t*_Pd_). The simulated spectra are calculated from Pd and Pd_2_Ge reference spectra, assuming an ideal Pd/Pd_2_Ge bilayer system. (c) Valence electron spectra, extracted from XPS survey scans. The gray background indicates the region above the Fermi level. The dashed lines indicate peak positions which are characteristic for Pd_2_Ge and Pd.

The XRR signal shows the SLD profile, but does not give direct information about the positions and chemical interaction of the elements. To obtain this complementary information, the Pd/Pd_2_Ge interface was studied by thickness-dependent postgrowth XPS measurements, shown in Figure 7a. With increasing *h*_Pd_, the Pd 3d_5/2_ XPS peak evolves from a symmetric peak at 336.35 ± 0.1 eV (*t*_Pd_ ≤ 90 s) to an asymmetric peak at 335.1 ± 0.1 eV (taking into account the expected systematic error). The initial peak position is attributed to Pd_2_Ge; the final position and peak shape indicate metallic Pd. The transition from Pd_2_Ge to Pd is also confirmed by the evolution of the valence spectra. With increasing Pd thickness, the shape of the spectra changes, the maximum shifts closer to the Fermi energy, and the density of populated states at the Fermi energy increases (Figure 7c).^48−50^

A model calculation of the thickness-dependent XPS spectra was performed,^51^ assuming an abrupt interface between Pd_2_Ge and Pd (Figure 7b) (for details, see the Supporting Information). The experimental spectra after deposition of *h*_Pd_ = 1.80 nm (*t*_Pd_ = 90 s) and *h*_Pd_ = 9.00 nm (*t*_Pd_ = 450 s) were used as reference spectra for bulk Pd and a Pd_2_Ge interlayer. The obtained germanide thickness  nm (calculated from *h*_Pd_) is close to  nm and  nm (systematic error ±0.1 nm), but underestimates the real value slightly due to the relatively large thickness steps between subsequent XPS spectra. The thickness-dependent change of the calculated XPS signal agrees well with the experimental observations, supporting the assumption of an abrupt Pd/Pd_2_Ge interface. Compared to Pd/Pd_2_Ge, the intermixing at the Pd/Pd_2_Si interface is stronger. The resulting peak shift extends over a larger thickness range and could not be reproduced with the above-described model.^37^

Combining the results of the real-time and postgrowth measurements, we have confirmed the formation of a highly oriented, textured Pd/Pd_2_Ge bilayer during Pd deposition on a-Ge. The XRD results indicate that each crystallite consists of a perfectly aligned epitaxial Pd_2_Ge(111)/Pd(111) bilayer. A comparatively sharp interface between Pd and Pd_2_Ge, as expected for an epitaxial system, is also confirmed by XPS. In the following, we will study the impact of the as-grown Pd/a-Ge system on the structure formation during annealing.

### 3.4 Solid-State Reaction during Annealing

**Figure 8:**
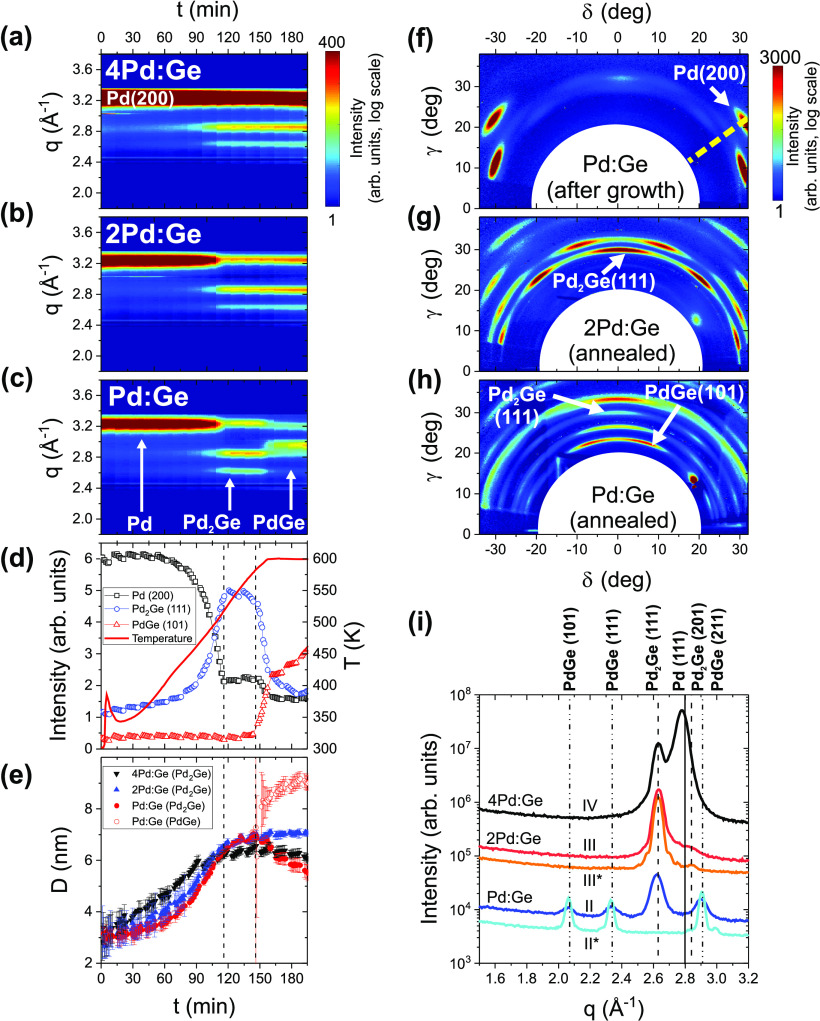
(a–c) XRD signal during annealing of Pd/Ge films with different Pd:Ge ratio (samples II–IV). (d) Intensity evolution during annealing of Pd:Ge (sample II), extracted from characteristic Bragg peaks of Pd, the first-forming Pd_2_Ge, and the second-forming PdGe phase. The reference data for the temperature evolution at the sample surface are shown as red line. (e) Grain size evolution of Pd_2_Ge and PdGe during annealing (samples II–IV). (f–h) XRD maps before and after annealing of Pd/Ge films (samples II and III). The radial scans at χ = 54°, shown in panels a–c, were extracted along the dashed line in panel f. The labeled XRD peaks are used for the quantitative analysis shown in panel d. (i) Ex situ XRD scans along the surface normal (χ = 0°, i.e., *q* = *q*_*z*_), for the samples II–IV (studied in real-time at the synchrotron), and for the reference samples II* and III*. For comparison, the data shown in panel i are plotted with a vertical offset.

For a systematic study of the thermal evolution, bilayers with different Pd and Ge reservoirs but the same expected Pd_2_Ge thickness of 13.5 nm (samples II–IV) were deposited and subsequently annealed up to *T* = 600 K. The experimental results are summarized in Figure 8, in which panels a–e give an overview over the time-dependent phase evolution, panels f–h show examples of reciprocal space maps before and after annealing, and panel i compares ex situ XRD scans at χ = 0°, obtained after annealing. The respective figures and observations will be detailed in the following.

Figure 8a–c gives an overview of the time-dependent phase evolution for nominally stoichiometric (atomic ratios 2Pd:Ge and Pd:Ge) and overstoichiometric bilayer systems (4Pd:Ge). The reference temperature at the sample surface is plotted as a red line in Figure 8d. Up to *t* ≈ 150 min, the evolution of the XRD pattern at χ = 54° is identical for all samples. The first forming phase is Pd_2_Ge. Only for Pd:Ge (sample II), the subsequent formation of PdGe is observed (Figure 8c). Postgrowth XRD measurements along the surface normal (Figure 8i) confirm coexisting PdGe and Pd_2_Ge phases for the unclamped sample II, and a complete transformation of Pd_2_Ge to PdGe for the clamped sample II*, likely due to a better thermal contact. Only for 4Pd:Ge (sample IV), a large amount of unreacted Pd is found after annealing.

The XRD maps in Figure 8f–h summarize the characteristic textures before and after annealing. To enlarge the features close to the surface normal, the data are plotted in angular space. Initially, all samples feature the above-described Pd_2_Ge[111] interlayer ( nm) covered by Pd[111] (Figure 8f). The intensity of all Bragg peaks related to Pd_2_Ge[111] increases during first-phase formation, as shown in Figure 8g for 2Pd:Ge. This indicates that the as-deposited germanide interlayer acts as a seed layer for the thermally induced Pd_2_Ge crystal growth. After second-phase formation [Figure 8c, sample II (Pd:Ge)], a reduced Pd_2_Ge[111] fraction coexists with PdGe[201] and other, less intense PdGe textures (Figure 8h). With 21 ± 2°, PdGe[201] is less textured than Pd_2_Ge[111].

The quantitative analysis of the diffraction patterns provides further information about the reaction process: Figure 8d shows the intensity evolution of the selected peaks during annealing of Pd:Ge (sample II). Pd_2_Ge(111) at χ = 15° represents the Pd_2_Ge phase and PdGe(101) at χ = 18° the PdGe phase. The Pd(200) peak at χ = 54° seems to decrease in two steps. Even after complete reaction to Pd_2_Ge, the intensity measured at the Pd(200) position cannot become 0 since the Pd peak overlaps with the scattered intensity of all phases. The Pd_2_Ge intensity increases exponentially up to *t*_1_ = 116 min (*T* = 389 K), accompanied by a decrease of the Pd scattering. It is worth noting that similar evolutions were also observed for 2Pd:Ge (sample III) and 4Pd:Ge (sample IV). These findings confirm that the Pd_2_Ge phase forms continuously at the expenses of Pd (and a-Ge). As expected, the reaction of all bilayers stops at the same time (Figure 8a–c), indicating that the respective reaction-limiting element (Pd for Pd:Ge, Ge for 4Pd:Ge, and both elements for 2Pd:Ge) is consumed. Only for Pd:Ge, another phase (PdGe) begins to form at *t*_2_ = 146 min (*T* = 583 K), at the expense of Pd_2_Ge.

The phase formation is accompanied by a grain size evolution, plotted in Figure 8e. The size of the Pd_2_Ge (samples II–IV) and PdGe (sample II) crystallites was determined from the fwhm of the Bragg peaks at χ = 15°. During first-phase formation, the Pd_2_Ge grain size (measured at χ = 15°) increases slowly up to approximately 7 nm for all samples. At the onset of PdGe formation, the PdGe grain size increases abruptly to about 8 nm, followed by a slow increase to 9 nm during further annealing, while the average Pd_2_Ge grain size decreases slowly.

The germanide grain size measured during annealing is smaller than expected, considering the deposited amount of Pd and Ge. This can be explained with the tilted orientation of the grains accessible at grazing incidence, and by an incomplete second-phase formation (sample II). For the completely reacted samples II* and III*, the average PdGe and Pd_2_Ge grain size along the surface normal is close to the expected value, but the tilted Pd_2_Ge grains are significantly smaller. This is in agreement with a competitive growth mechanism favoring the grains oriented along the surface normal. Nevertheless, even if the real-time data probe slightly misoriented grains, they provide valuable information about the grain size evolution during annealing. Quantitative information about the χ-dependent grain size is provided in Table S2 (Supporting Information).

In summary, we have confirmed the phase sequence, first Pd_2_Ge then PdGe, reported in the literature. After complete reaction, the well-oriented Pd_2_Ge[111] and PdGe[201] crystallites extend over the entire germanide layer thickness. The stronger Pd_2_Ge texture after solid-state reaction indicates that the as-deposited germanide interlayer acts as a seed layer for competitive, thermally induced Pd_2_Ge crystal growth. In contrast to Pd_2_Ge, PdGe does not form on a template and has therefore a lower degree of orientation. Nevertheless, the PdGe texture suggests an interface-related nucleation process for the second-forming phase. Assuming that the interface is sufficiently smooth, it can therefore impose a well-defined texture during growth and annealing of the metal/amorphous semiconductor interfaces. Conversely, an increasing interface roughness would affect both texture and phase formation, explaining contradicting reports about the phase evolution at the metal–semiconductor interface.

### 3.5 Chemical Composition and Morphology after Annealing

**Figure 9:**
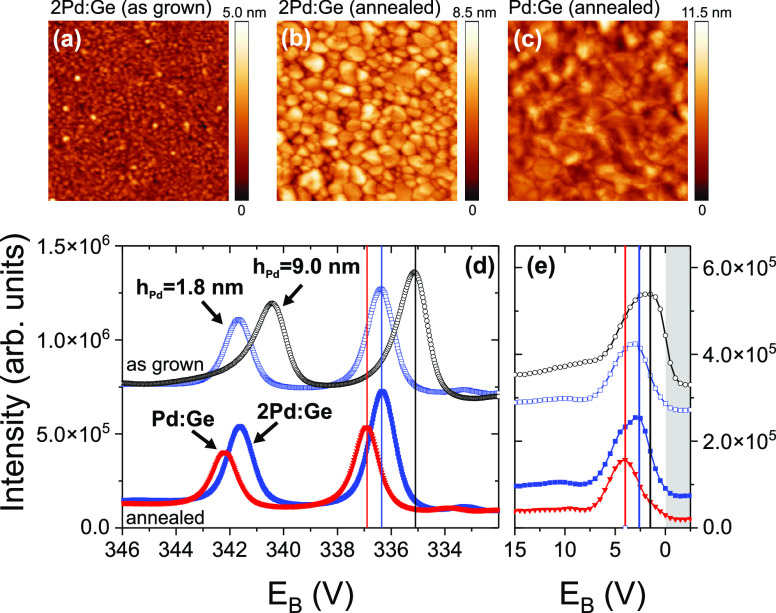
Noncontact AFM images (image size 500 × 500 nm^2^) after (a) deposition and (b, c) annealing of Pd/a-Ge layer systems with different Pd:Ge ratios (samples II* and III*). (d) Corresponding Pd 3d XPS spectra after annealing. The spectra are compared to reference spectra at *h*_Pd_ = 1.8 nm (blue circles), and *h*_Pd_ = 9.0 nm (black circles, sample II* before annealing), i.e., after the expected formation of a 2.7 nm Pd_2_Ge interlayer, and after growth of ∼7 nm Pd on top of the interlayer. (e) Corresponding valence band spectra, extracted from XPS survey scans. The energy range above the Fermi level is indicated by a gray background. For comparison, the XPS data are plotted with a vertical offset.

Complementary to the in situ XRD measurements, the morphology and composition changes caused by the annealing were studied by UHV noncontact AFM and XPS. The AFM and XPS results of the samples II* (Pd:Ge) and III* (2Pd:Ge) are summarized in Figure 9. Additional images with a larger scan range are provided in the supporting information (Figure S2). For the as-deposited films, the lateral features have a typical size of 20–30 nm (shown only for 2Pd:Ge). After annealing, the 2Pd:Ge and Pd:Ge samples show quite different morphologies. The feature size increases to 40–60 nm for the annealed 2Pd:Ge film. Only few large objects with sizes of about 100 nm are observed. For the annealed Pd:Ge film, however, the grain boundaries are less obvious, and the lateral feature size is of the order of 100 nm. The root-mean-square roughness of samples Pd:Ge and 2Pd:Ge was determined from several noncontact AFM images with a size of at least 1000 × 1000 nm^2^, taken at different sample positions. After deposition, the values were in the range 0.35–0.45 nm. During annealing, the roughness increased by a factor of 2–3. The AFM results indicate a high surface and grain boundary mobility of the germanides. However, the images reflect only the state after the complete annealing cycle and cannot be directly related to the phase transformation.

The germanide formation affects the chemical interaction of Pd and Ge, which is reflected by the XPS core level peaks and the valence band. Figure 9d compares the Pd 3d XPS spectra of annealed (filled symbols) and as-deposited Pd/a-Ge samples (open symbols). The corresponding valence band spectra are shown in Figure 9e. The XPS spectrum of the annealed 2Pd:Ge film (blue squares, sample III*) is identical to the spectrum of the interlayer (blue squares, open), confirming the full conversion of the Pd/a-Ge film to Pd_2_Ge. The Pd 3d_5/2_ peak of the annealed Pd:Ge film (sample II*) is found at 336.9 eV and the corresponding valence band maximum at 4 eV. In agreement with the ex situ XRD results, we attribute both features to PdGe. The XPS peaks of both germanides are nearly symmetric, in contrast to the asymmetric shape of metallic Pd. From PdGe via Pd_2_Ge to Pd, the valence band changes its shape and shifts to the Fermi energy. This evolution is very similar to observations for the PdSi, Pd_2_Si, and Pd series.^49^ For Pd–Ge alloys with 35–73% Pd content, Suzuki et al. reported a double peak structure of the valence band.^50^ Even with our low experimental resolution, this structure is visible for the annealed 2Pd:Ge sample, but not for the Pd_2_Ge interlayer after 90 s. We assume that the peak shape is affected by the composition gradient at the Ge–Pd_2_Ge interface, since Suzuki et al. reported a decreasing peak distance for increasing Ge content.^50^

## 4 Discussion

### 4.1 Structure Formation during Deposition

**Figure 10:**
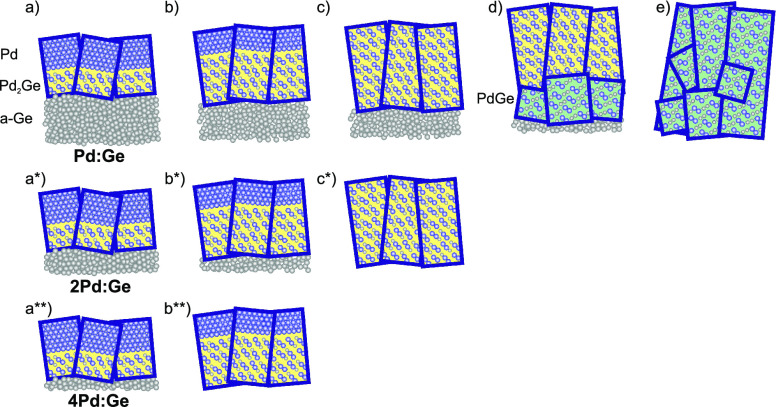
Structure evolution during growth and annealing, sketched for the experimentally studied atomic ratios Pd:Ge (a–e), 2Pd:Ge (a*–c*), and 4Pd:Ge (a**–b**). Irrespective of the Pd fraction, an oriented Pd_2_Ge interlayer forms during deposition at the interface between Pd and a-Ge (a, a*, a**). During annealing, the individual Pd_2_Ge grains grow (b, b*, b**) up to the complete consumption of a-Ge (b**, c*) and/or Pd (c, c*). If the Pd reservoir is empty but unreacted a-Ge is still available (c), a less oriented PdGe layer nucleates at a sufficiently high temperature at the Pd_2_Ge/a-Ge interface (d) and grows up to the depletion of the a-Ge reservoir (e).

Based on the experimental observations reported above, Figure 10a,a*,a** summarizes our understanding of the polycrystalline, highly oriented film formed during Pd deposition on a-Ge. The XRD results indicate that each crystallite consists of a perfectly aligned, epitaxial Pd_2_Ge(111)/Pd(111) bilayer. A Pd_2_Ge interlayer of 4–5 nm was also reported in ref (43). Radulescu et al.^52^ have reported a slightly higher energy barrier *E*_B_ = 1.12 eV for Pd_2_Ge growth compared to *E*_B_ = 1.03 eV for Pd–Ge interdiffusion. Our observations indicate that this barrier is related to the crystallization of Pd_2_Ge. In agreement with our model for Pd/a-Si growth,^37^ we propose that the crystallization decreases the effective Pd and Ge transport through the interlayer. This would correspond to an increase of the effective energy barrier for the atomic transport, which would abruptly slow down the Pd_2_Ge formation (in agreement with diffusion-limited growth), and allow for Pd grain growth.

Using a combination of real-time curvature, synchrotron XRD and XRR, and postgrowth XPS experiments, we have confirmed the isostructural interface formation of Pd/a-Ge and Pd/a-Si. Both interfaces form in four steps: (1) A mixed, amorphous, or nanocrystalline Pd_2_Ge (Pd_2_Si) interlayer grows. (2) The interlayer crystallizes with [111] texture. (3) A coherent Pd(111) layer nucleates after interlayer formation (2.9 ± 0.1 nm Pd_2_Ge or 3.7 nm Pd_2_Si). (4) The (locally) epitaxial Pd layer nucleates and evolves via competitive crystal growth.

Independent of the semiconductor, the tensile maximum associated with the interlayer crystallization appears at *t*_Pd_ ∼ 100 s. This position coincides with the formation of an abrupt Pd/Pd_2_Ge interface. The Pd/Pd_2_Si interface appears later and is less abrupt. After relaxation of the tensile peak, the stress state is more compressive for Pd/a-Ge than for Pd/a-Si. We propose that these effects are related. Due to the larger atomic radius of Ge compared to Si, a larger lattice mismatch occurs at the Pd/Pd_2_Ge interface. The compressive strain due to lattice mismatch counteracts the tensile strain after interlayer crystallization. It seems that the small lattice mismatch facilitates the continued growth of coexisting Pd and Pd_2_Si phases, increasing the interlayer thickness and slowing down the relaxation of the tensile stress. The large lattice mismatch of Pd/Pd_2_Ge favors a minimization of the interface area, i.e., the formation of an abrupt interface. A priori, a Pd/a-Ge contact with such a sharp interface should be preferable for producing metal/semiconductor contacts with predictable properties. However, the lattice strain between metal and interlayer is expected to reduce the long-term stability of the contact and, thus, the device performance. Therefore, even in the case of laterally isotropic, polycrystalline systems, possible epitaxial strains between the metal and reactively formed interlayer should be considered.

### 4.2 Structure Formation during Annealing

We have reported on the structural evolution during growth and annealing of Pd/a-Ge bilayers with different Pd:Ge atomic ratios. In agreement with earlier ex situ^53−55^ and more recent in situ X-ray studies,^42−45^ our experiments confirm Pd_2_Ge as the first-forming and PdGe as the second-forming phase. The reactive growth of Pd_2_Ge is already observed during deposition at room temperature. During annealing, an interface-mediated solid-state reaction occurs. The diffusion at both interfaces (i.e., Pd_2_Ge/a-Ge and Pd/Pd_2_Ge) is sufficiently large to provide a continuous supply for Pd_2_Ge formation. We have studied bilayers with different Pd fractions *x* = 0.5, 0.66, and 0.8. Only for *x* = 0.5, the PdGe formation was observed above a critical temperature *T* = 583 K. The onset of PdGe formation at a specific temperature indicates a nucleation barrier for the phase formation. According to Radulescu et al.,^52^ the transition temperature depends on layer thickness and annealing rate. The concept of nucleation barriers with thermodynamic and kinetic contributions was successfully used by several authors, e.g., recently by van Stiphout et al.^25^ Further experiments with different annealing rates and layer thicknesses would be required to disentangle both contributions.

Beyond the direct observation of different phases, our results give insight into the microstructure evolution during phase formation. The structure evolution during annealing is sketched in Figure 10. Figure 10a–e summarizes the structural evolution of Pd:Ge, Figure 10a*–c* the evolution of 2Pd:Ge, and Figure 10a**,b** the evolution of 4Pd:Ge. All samples follow the same reaction path but stop at different stages, depending on the available material. In Figure 10a,a*,a**, after deposition, irrespective of the Pd fraction, a Pd_2_Ge(111) interlayer forms between the a-Ge and a highly oriented Pd(111) layer (Figure 10b,b*,b**). The Pd_2_Ge crystallites of this seed layer grow continuously during annealing. This indicates that the diffusion through the interlayer is the limiting factor for Pd_2_Ge growth. The solid-state reaction of Pd and Ge proceeds up to the full consumption of either Ge (Figure 10 b**) or Pd (Figure 10c,c*) at *t*_1_. For 2Pd:Ge and 4Pd:Ge, the equilibrium phase composition is reached, and structure evolution ends. For Pd:Ge, a second solid-state reaction takes place at *t*_2_, with PdGe crystallites nucleating and growing at the interface between Pd_2_Ge and a-Ge (Figure 10d). With the sample Pd:Ge, we have probed the reaction until depletion of the a-Ge reservoir (Figure 10e). The PdGe texture indicates a nucleation at the Pd_2_Ge/a-Ge interface. However, due to the different atomic arrangement in both germanides, the second phase is less well oriented and forms several coexisting textures. After complete reaction, the well-oriented Pd_2_Ge (after first-phase formation) or PdGe crystallites (after second-phase formation) extend over the entire germanide layer thickness. Note that our experiments do not provide information about the germanide growth direction after initial nucleation (up/down with respect to the interface). Taking into account the results of Perrin Toinin et al.,^43^ the simultaneous growth in both directions seems likely.

The case of a-Ge excess, missing in our systematic approach, was studied by Geenen et al.^42^ The initial stages of the process agree with our observation: After complete PdGe formation, the reaction process stopped, as observed for Pd:Ge (Figure 10e). Geenen et al.^42^ and Perrin Toinin et al.^56^ reported two transition temperatures, 473 and 389 K for Pd_2_Ge and 573 and 503 K for PdGe, respectively. The abrupt onset of PdGe formation agrees with our observation. The absence of a critical temperature for Pd_2_Ge growth has not yet been reported, likely limited by the signal-to-noise ratio of the XRD signal.

A high grain boundary density and a low degree of orientation are known to increase the contact resistance. A competition between interface and bulk nucleation, as observed for PdGe, is therefore undesired. The nucleation at the interface, as observed for Pd_2_Ge, reduces the number of possible nucleation sites and offers a starting point for optimizing the grain size.

The crystal growth during annealing of Pd/crystalline Ge (c-Ge) is known to be similar, but not identical, to the structure formation of Pd/a-Ge.^42^ Independent of the long-range order of the Ge surface, the nucleation at the interface seems to be preferred, as evidenced by the observation of epitaxial and axiotaxial germanide textures after annealing. Therefore, the formation of a seed layer during deposition, independent of the Ge crystallinity, seems to be likely. For c-Ge, this might require an intermediate amorphization step.^25^ However, without further experimental evidence we cannot conclude on the texture, crystallinity, and growth mechanism of such a seed layer.

### 4.3 Structure Evolution and Pd/a-Ge Contact Characteristics

**Figure 11:**
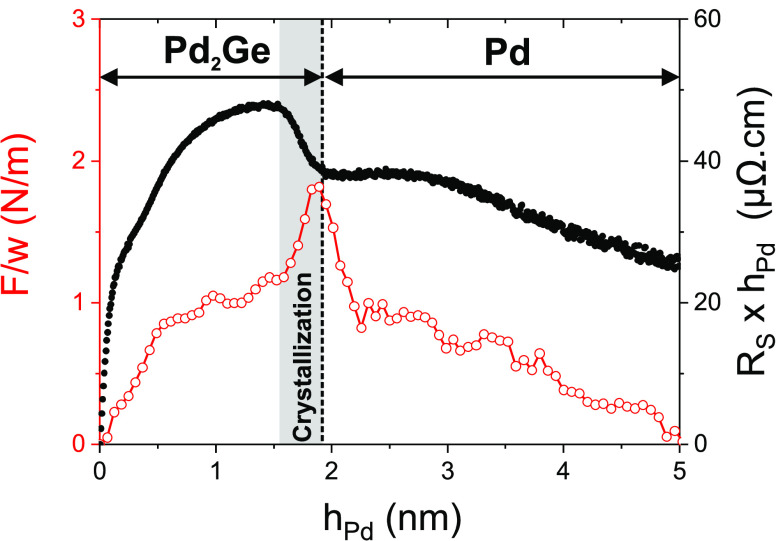
Real-time resistivity (black dots, right axis) and stress measurements (red circles, left axis) during deposition. Partially reproduced with permission from ref (57). Copyright 2020 MDPI.

With the detailed picture of the structure formation during Pd deposition on a-Ge, it is now possible to understand the resistivity evolution of a Pd/a-Ge contact during deposition. Figure 11 shows real-time resistivity (black dots, right axis), which is equivalent to the product of sheet resistance *R*_S_ and *h*_Pd_, and stress data (red circles, left axis) measured during early growth stages. The data were originally published in ref (57). The deposition was done in a different growth chamber, using a 4 times higher Pd deposition rate (*F*_Pd_ = 0.076 nm/s). Nevertheless, the stress evolution during deposition is nearly identical to the results reported here. Thus, the resistivity increase below 1 nm Pd can be related to the early stages of interface formation. During further deposition, the resistivity increase slows down. The sudden crystallization of the Pd_2_Ge interlayer, visible as a tensile peak, is clearly identified as origin of the resistivity drop around h_Pd_∼1.5–2.0 nm. The much slower resistivity decrease during later stages of Pd deposition is related to a decreasing defect density, in agreement with the slowly improving Pd(111) texture. Interestingly, the interface between two polycrystalline metallic layers, Pd_2_Ge and Pd, does not have a visible impact on the resistivity, likely due to the coherent interface within the grains.

## 5 Conclusions

Using noninvasive, surface-sensitive in situ X-ray and UHV surface characterization methods, we have obtained a detailed understanding of the structure formation during growth and subsequent annealing of Pd/a-Ge and could unveil the interplay between structure and electronic properties during formation of ultrathin Pd/a-Ge contacts.

For Pd deposition on a-Ge, we have found real-time evidence for a coherent, locally epitaxial growth, which is extremely difficult to access for laterally isotropic systems. We have confirmed the isostructural interface formation during deposition of Pd on a-Si and a-Ge. The analogy between both systems extends to the stress evolution and texture formation during deposition. Our results show that the as-deposited interface and the available material reservoir play an important role for the temperature-induced microstructure evolution during first- and even second-phase formation. The phase formation and microstructure evolution during annealing follow a general scheme but stop at different stages, depending on the available Pd and Ge reservoir. The interface-mediated formation of Pd_2_Ge and PdGe takes place via different mechanisms, comparable to homo- and heteroepitaxy.

Our real-time study gives access to the interplay between structure and electronic properties during contact formation. Furthermore, it reveals critical issues for the long-term stability of nanoscale devices, such as intermixing and lattice strain due to interlayer formation. Lastly, it provides information about phase nucleation mechanisms, enabling thus a knowledge-based interface design and opening new ways to improve the contact performance.

## Supporting Information Available

Detailed information about the simulated real-time XRD data; calculation of the thickness-dependent XPS spectra; angle-dependent germanide grain size evolution; and additional AFM images (PDF)
