# Supplementary material for: In Situ Study of the Interface-Mediated Solid-State Reactions during Growth and Postgrowth Annealing of Pd/a-Ge Bilayers
Source: ACS Appl Mater Interfaces. 2023 Feb 15;15(8):11268–80. doi: 10.1021/acsami.2c20600 (PMC9983571; doi:10.1021/acsami.2c20600)
Supplement: Supplementary file 1 — am2c20600_si_001.pdf [file am2c20600_si_001.pdf]

Supporting Information: In situ study of the interface-mediated solid-state reactions during growth and post-growth annealing of Pd/a-Ge bilayers

Bärbel Krause,[*,†] Gregory Abadias,[‡] David Babonneau,[‡] Anny Michel,[‡] Andrea Resta,[¶] Alessandro Coati,[¶] Yves Garreau,[¶,§] Alina Vlad,[¶] Anton Plech,[†] Peter Wochner,[‖] and Tilo Baumbach[†,⊥]

†*Institut für Photonenforschung und Synchrotronstrahlung (IPS), Karlsruher Institut für Technologie, D-76021 Karlsruhe, Germany*

‡*Institut PPrime, Département Physique et Mécanique des Matériaux, UPR 3346 CNRS, Université de Poitiers, SP2MI, TSA 41123, Cedex 9, 86073 Poitiers, France*

¶*Synchrotron SOLEIL, L'Orme des Merisiers, Départementale 128, 91190 Saint Aubin, France*

§*Laboratoire Matériaux et Phénomenes Quantiques, Université Paris Cité, Paris, France*

‖*Max Planck Institute for Solid State Physics, Heisenbergstraße 1, D-70569 Stuttgart, Germany*

⊥*Laboratorium für Applikationen der Synchrotronstrahlung (LAS), Karlsruher Institut für Technologie, D-76021 Karlsruhe, Germany*

E-mail: baerbel.krause@kit.edu

1 Crystalline structures used for the data analysis

Table S1: Relevant crystalline phases of the material systems Pd-Ge and Pd-Si, according to the inorganic crystal structure database (ICSD). The unit cell parameters a, b, c, the volume of the unit cell, V, and the number of molecular units, Z, are given.

Material	Space group	Crystal family	a (Å)	b (Å)	c (Å)	V (Å^3)	Z	ICSD entry
Ge	227	cubic	5.6579	5.6579	5.6579	181.12	8	636527
Pd_2Ge	189	hexagonal	6.712	6.712	3.408	132.96	3	53879
PdGe	62	orthorhombic	6.259	5.782	3.481	125.98	4	76624
Si	227	cubic	5.4305	5.4305	5.4305	160.15	8	51688
Pd_2Si	189	hexagonal	6.496	6.496	3.433	125.457	3	43209
PdSi	62	orthorhombic	6.1534	5.6173	3.3909	117.208	4	15016
Pd	225	cubic	3.8907	3.8907	3.8907	58.8957	4	53879

2 Calculation of the real-time XRD data

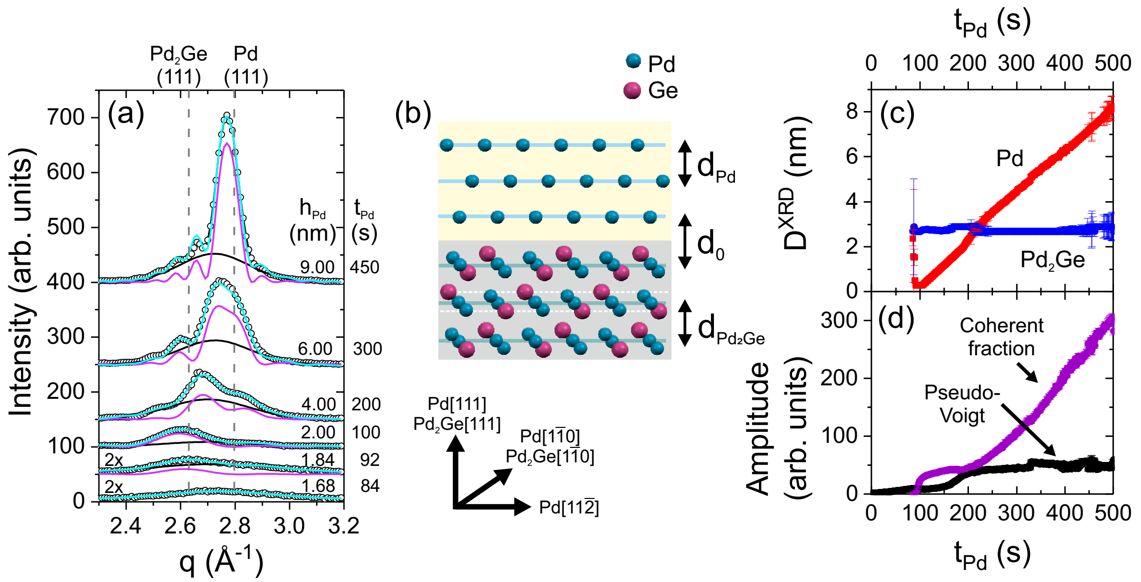

Figure S1: (a) Experimental data (circles, sample I) and simulated XRD curves (blue lines) at selected t_{Pd}. Curves corresponding to different h_{Pd} (t_{Pd}) are plotted with vertical offset). For early growth stages, the intensity is multiplied by 2. The simulation assumes a coherent superposition of Pd_2Ge and Pd scattering after crystallization (violet line). A Pseudo-Voigt function represents the broad intensity distribution during early growth stages and the incoherent contribution after crystallization (black line). Vertical dashed lines indicate the expected Pd(111) and Pd_2Ge(111) peak positions. (b) To facilitate the reading, we repeat here the Pd/Pd_2Ge bilayer model shown also in Fig. 6 (c). (c) Pd and Pd_2Ge thickness used for the calculation of the coherent fraction. (d) Fitted amplitude of both contributions.

Fig. S1 (a) shows vertically offset line scans, extracted at different t_{Pd} from the real-time XRD maps (Fig. 6). The experimental data are denoted by symbols, the fit curves are shown as lines. For $t_{Pd} > 90$ s, two scattering contributions were assumed: a coherent, kinematic scattering from perfectly aligned Pd(111) and Pd_2Ge(111) crystallites [violet line in Fig. S1 (a)], and an incoherent contribution described by a Pseudo-Voigt function (Gaussian fraction 0.9, black line). The Pseudo-Voigt function was also used to describe the broad, Lorentzian intensity distribution for $t_{Pd} \leq 90$ s. The sum of both compoments is shown as blue line. The amplitudes of the coherent and incoherent scattering contribution were allowed to vary freely. All other parameters were varied in a narrow range around the

expected values (bulk lattice parameters and the time-dependent layer thickness determined by XRR). For clarity, the time-dependent layer thickness (which was forced to stay close to the value expected from the XRR analysis) is plotted in Fig. S1 (c). The fitted amplitudes are shown in Fig. S1 (d). The incoherent fraction increases linearly before crystallization. After onset of crystallization, the coherent fraction dominates the intensity distribution. For $100 \lesssim t_{Pd} \lesssim 200$ s, the amplitude of the coherent fraction is determined by the Pd_2Ge layer and nearly constant. Then, Pd becomes dominant and the coherent amplitude increases with increasing Pd layer thickness.

3 Calculation of the thickness dependent XPS spectra

The model calculation of the thickness-dependent XPS spectra was performed,[S1] assuming an abrupt interface between Pd_2Ge and Pd. The electron inelastic mean free paths (IMFP) λ in Pd and Pd_2Ge were taken from the NIST IMFP Database, using the predictive formula of Gries.[S2] Neglecting the contribution of atoms located directly at the interface, which do not have the bulk chemical environment of Pd or Pd_2Ge, the thickness-dependent XPS intensity can be written as sum of the Pd_2Ge and Pd contributions:

$$I(t) = I_{Pd-Ge}(t) + I_{Pd-Pd}(t). \tag{1}$$

Using the XPS spectrum at $t_{Pd} = 450$ s as reference for the signal of an infinite Pd layer, I^{∞}_{Pd-Pd}, the Pd 3d signal of a thin Pd layer with $\lambda_{Pd} = 1.17$ nm and thickness D_{Pd} is

$$I_{Pd-Pd}(t) = I^{\infty}_{Pd-Pd}\left(1 - e^{-\frac{D_{Pd}(t)}{\lambda_{Pd}}}\right). \tag{2}$$

The signal of the buried Pd_2Ge interlayer with the IMFP $\lambda_{Pd_2Ge} = 1.4$ nm is corrected by an additional absorption factor, taking into account the electron scattering in the Pd layer:

$$I_{Pd-Ge}(t) = I^{\infty}_{Pd-Ge}(t) \left(1 - e^{-\frac{D_{Pd_2Ge}(t)}{\lambda_{Pd_2Ge}}} \right) e^{-\frac{D_{Pd}(t)}{\lambda_{Pd}}} . \quad (3)$$

I^{∞}_{Pd-Ge} was calculated from the Pd_2Ge signal at $t_{Pd} = 90$ s since the subsequent spectrum at $t_{Pd} = 100$ s (expected $D_{Pd_2Ge} = 3.0$ nm) revealed a small peak shift. The XPS scans probe the sample surface at much larger thickness intervals than the real-time methods. Therefore, the Pd_2Ge thickness after 90 s Pd deposition ($D^{XPS}_{Pd_2Ge} = 2.72$ nm) likely underestimates the interlayer thickness, but is still close to $D^{XRD}_{Pd_2Ge} = 3.0 \pm 0.2$ nm and $D^{XRR}_{Pd_2Ge} = 2.86 \pm 0.1$ nm.

4 Grain size

Table S2: Grain size in nm, determined from the $Pd_2Ge(111)$ and PdGe(101) Bragg peaks collected during or after thermal annealing. The data at $\chi = 0°$ (along the surface normal) were extracted from the *ex situ* XRD scans shown in Figure 8 (i), the data at $\chi = 15°$ were extracted from the real-time data at $t = 195$ min. The expected thickness, calculated from the available material and the bulk densities, is given for comparison.

	Sample	germanide grain size (nm)			
		Pd_2Ge ($\chi = 0°/15°$)		PdGe ($\chi = 0°/15°$)	
Pd:Ge	II	8.9 ± 0.05	5.4 ± 0.5	7.7 ± 0.7	9.5 ± 0.5
	II*			16.7 ± 0.5	
2Pd:Ge	III	10.9 ± 0.1	7.0 ± 0.2		
	III*	12.9 ± 0.1			
4Pd:Ge	IV	10.6 ± 0.4	6.1 ± 0.3		
expected		13.5		18	

5 Additional AFM images

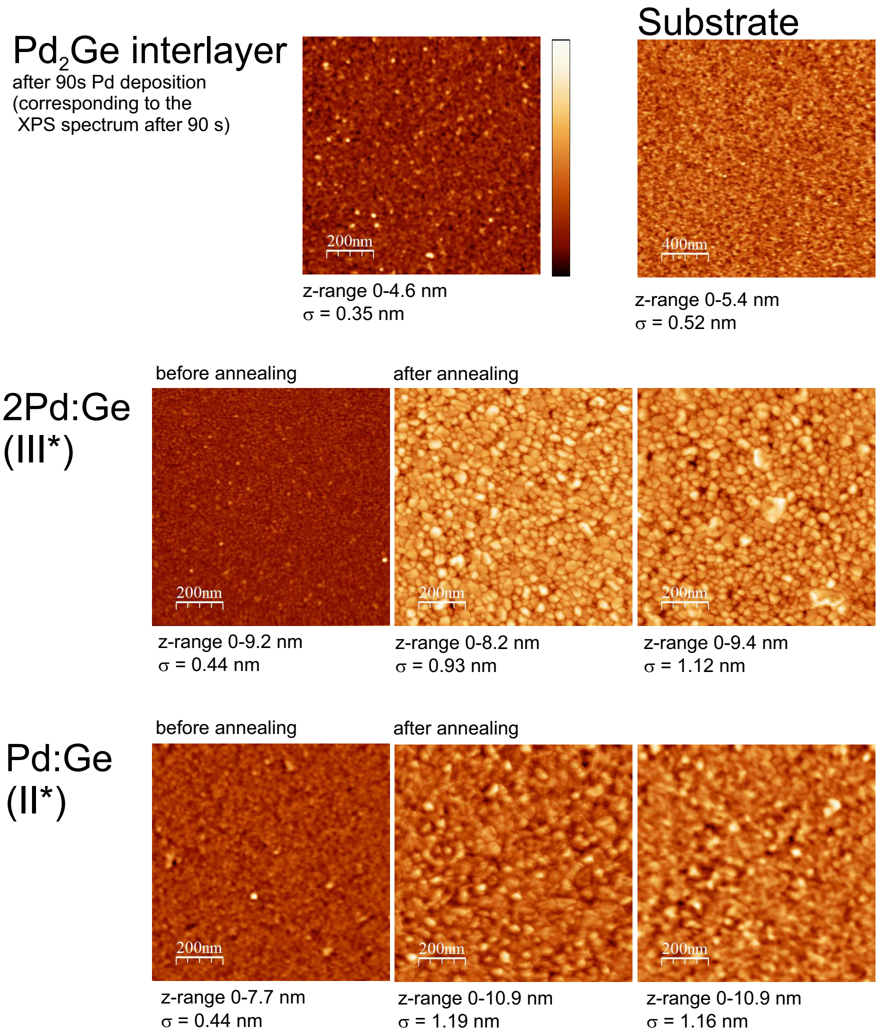

Figure S2: AFM images before deposition, after Pd_2Ge interlayer formation (90s Pd deposition), and before and after annealing of 2Pd:Ge (sample III*) and Pd:Ge (sample II*). All images use the same color scale. The respective z range and RMS roughness are indicated below each image.

References

(S1) Briggs, D.; Grant, J. T. *Surface Analysis by Auger and X-ray Photoelectron Spectroscopy*; IM Publications and SurfaceSpectra Limited, 2003.

(S2) Powel, C. J.; Jablonski, A. *NIST Electron Inelastic-Mean-Free-Path Database, Version 1.2, SRD71*; National Institute of Standards and Technology, Gaithersburg, MD, 2010.
